# Exploiting Size-Dependent Drag and Magnetic Forces for Size-Specific Separation of Magnetic Nanoparticles

**DOI:** 10.3390/ijms160820001

**Published:** 2015-08-21

**Authors:** Hunter B. Rogers, Tareq Anani, Young Suk Choi, Ronald J. Beyers, Allan E. David

**Affiliations:** 1 Department of Chemical Engineering, Auburn University, 212 Ross Hall, Auburn, AL 36849, USA; E-Mails: hunterrogers2014@u.northwestern.edu (H.B.R.); tba0008@tigermail.auburn.edu (T.A.); yzc0036@auburn.edu (Y.S.C.); 2 Auburn University MRI Research Center, Auburn, AL 36849, USA; E-Mail: rjb0018@auburn.edu

**Keywords:** field-flow fractionation, iron oxide nanoparticles, size separation, magnetic nanoparticles, nanomedicine

## Abstract

Realizing the full potential of magnetic nanoparticles (MNPs) in nanomedicine requires the optimization of their physical and chemical properties. Elucidation of the effects of these properties on clinical diagnostic or therapeutic properties, however, requires the synthesis or purification of homogenous samples, which has proved to be difficult. While initial simulations indicated that size-selective separation could be achieved by flowing magnetic nanoparticles through a magnetic field, subsequent *in vitro* experiments were unable to reproduce the predicted results. Magnetic field-flow fractionation, however, was found to be an effective method for the separation of polydisperse suspensions of iron oxide nanoparticles with diameters greater than 20 nm. While similar methods have been used to separate magnetic nanoparticles before, no previous work has been done with magnetic nanoparticles between 20 and 200 nm. Both transmission electron microscopy (TEM) and dynamic light scattering (DLS) analysis were used to confirm the size of the MNPs. Further development of this work could lead to MNPs with the narrow size distributions necessary for their *in vitro* and *in vivo* optimization.

## 1. Introduction

Nanomedicine is a broad area of research focused on the utilization of nanomaterials for the diagnosis, treatment, and prevention of diseases [1]. Biomedical applications present a unique opportunity to create engineered nanomaterials with highly controlled properties and functions that are comparable in scale to biological molecules and structures [2]. This is especially relevant for the fields of biomimetic nanomaterials, targeted drug delivery systems, and diagnostic imaging agents [3,4]. In contrast to the ever-growing list of nanomaterials researched for medical applications, the number of technologies actually approved for clinical use is relatively small. This is, in part, due to an overall paucity of fundamental knowledge and a lack of understanding of how the physical and chemical properties of nanomaterials affect their interactions with biological systems, and the associated uncertainty in their safety and toxicity profiles [5].

It is known that the behavior of nanomaterials within biological environments, including their stability and biodistribution, is dependent on their chemical composition and physical properties, such as size and geometry [6]. For nanomaterials to achieve their full potential in clinical applications, the fundamental principles governing their physio-chemical properties and the effects of these properties on physiological processes must be determined. However, obtaining nanoparticles of homogenous composition, either by finely controlled synthesis or through separation processes, has proven to be challenging. Particle size, in particular, can have a significant effect on the fate of nanoparticles once introduced into the body. For example, it has been shown that nanomaterials smaller than 6 nm are filtered out by the kidneys while those larger than 200 nm are more avidly taken up by macrophages and found to accumulate within the liver and spleen [7,8,9]. The biodistribution and pharmacokinetics of nanomaterials can also be affected by disease states. With cancer, for example, tumors typically possess a leaky vasculature and altered lymphatic drainage that results in the enhanced permeability and retention (EPR) effect, which enables extravasation of nanomaterials into the tumor microenvironment, with particles of size less than 200 nm typically providing greatest tumor penetration [10,11].

Magnetic nanoparticles (MNPs), specifically those composed of iron oxide, have been studied extensively for use in a variety of applications in nanomedicine, especially in the area of drug delivery and biomedical imaging [12,13,14,15]. In fact, several MNP formulations have been approved for clinical application as imaging contrast agents (e.g., Feridex^®^/Endorem^®^, Resovist^®^/Cliavist^®^, and Sinarem^®^/Combidex^®^) but commercial production has been halted due to poor clinical performance [16,17,18]. An improved understanding of the structure-property-performance relationships of MNPs could significantly improve their clinical application. While synthesis of iron oxide nanoparticles having monodisperse diameters less than 30 nm is well established, the synthesis of larger, monodisperse, iron oxide nanoparticles has proved challenging and greatly limited their optimization for biomedical applications. [19,20,21]. Batch-to-batch variability in nanoparticle production and broad size distributions raise safety concerns for clinical application due to the dependence of pharmacokinetics and biodistribution on the particle’s physical and chemical properties [22]. In order to promote the clinical translation of MNPs, methods must be developed that either allow for synthesis of homogenous nanoparticles or enable their size-selective fractionation post-synthesis.

One potential solution to this problem is the use of magnetic field-flow fractionation (mFFF). This process, based on the separation of particles via the combined effects of the size-dependent drag and magnetic forces, was first reported in 1980 by Vickrey and Garcia-Ramirez who wrapped Teflon tubing around a small electromagnet in an attempt to separate nickel complexes of bovine serum albumin from a fluid [23]. While a number of studies in literature report the use of various mFFF approaches, these primarily focus on the use of mFFF for characterizing small volumes of micro- or nano-particles or for the separation of magnetic particles from non-magnetic materials [24,25,26]. Very little attention has been given to mFFF as a size-specific separation technique for magnetic nanoparticles, especially for nanoparticles within the size range relevant for biomedical applications. The use of high gradient magnetic separation (HGMS) has been used by several groups to separate colloid suspensions of large magnetic nanoparticles, but the focus was not necessarily on the sorting of a polydisperse MNP suspension into multiple samples having narrower and controlled size distributions [27,28]. Beveridge *et al.* did report the use of a differential magnetic catch and release as a size-selective separation technique; however, their work focused on magnetic nanoparticles with diameters less than 20 nm [29]. The focus of this study is on the separation of MNPs with a hydrodynamic diameter in the range of 50–400 nm, which have potential biomedical application. Several prototypes for MNP separation were tested and the polydisperse MNPs ultimately separated into fractions having a narrower size distribution. This ability to separate magnetic nanoparticles according to their size ultimately enables the fundamental studies required to advance the use of magnetic nanoparticles in medicine.

## 2. Theory

Magnetic nanoparticles introduced into a mFFF system experience drag and magnetic forces in proportion to particle size. Very small particles, such as those on the nanoscale, also exhibit random Brownian motion, which can significantly affect nanoparticle behavior. For small, spherical particles in a fluid possessing a small Reynolds number (*Re* < 1), the drag force *F_D_* can be described using Stokes drag, which is defined as:

(1) $$F_{D}=6\pi \eta rv$$

where η is the fluid viscosity, *r* is the hydrodynamic radius of the particle, and *v* is the fluid velocity [30]. The drag force is therefore directly proportional to the hydrodynamic radius of the particle. The magnetic force ${\vec{F}}_{M}$ experienced by a magnetic particle within an applied magnetic field $\vec{B}$ is given by the following equation:

(2) $${\vec{F}}_{M}=\left(\vec{m}\cdot \nabla \right)\vec{B}$$

where $\vec{m}$ is the magnetic moment of the particle, calculated using the equation:

(3) $$\vec{m}=\rho V\vec{M}$$

where ρ is the particle density, *V* is the volume of magnetic material in the particle, and $\vec{M}$ is the magnetization of the particle [31]. According to Equation (3), the magnetic force experienced by a particle is proportional to ~*r*^3^—increasing with size of the MNP, while the magnitude of the drag force (Equation (1)) is proportional to *r*. This dependence of the drag and magnetic forces on particle size provides a means to manipulate magnetic nanoparticles in a size-dependent manner.

As mentioned previously, for very small particles in a fluid, molecular collisions result in a source of diffusion known as Brownian motion. The Brownian diffusion length, $L_{D}$, traversed by a particle in two-dimensions over some time interval *dt* is approximated by the equation:

(4) $$L_{D}=\sqrt{4D\;dt}$$

where *D* is the particle-specific diffusion coefficient, defined as:

(5) $$D=\frac{k_{B}T}{6\pi \eta r}$$

where $k_{B}$ is the Boltzmann’s constant and *T* is the absolute temperature [32]. This relation shows that diffusion due to Brownian motion is also size-dependent and that the rate of diffusion decreases with increasing particle size. Taken together, these equations can be used to predict the movement of MNPs under the influence of drag and magnetic forces and Brownian motion.

## 3. Results and Discussion

### 3.1. Modeling the Effects of Drag and Magnetic Forces

**Figure 1:**
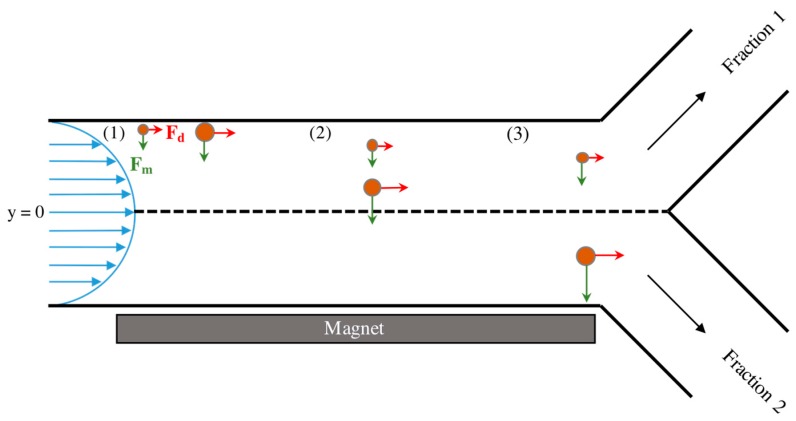
Illustration of the proposed experimental setup and the force balances experienced by two differently sized magnetic nanoparticles as they flow through the system. Green arrows represent the magnetic force (*F_M_*) and red arrows represent the drag force (*F_D_*). At position (**1**), the two MNPs are introduced to magnetic field in flow at the wall opposite the magnet. At a later time, the two particles reach position (**2**) and have separated from each other in the *y*-direction due to the increased magnetic force experienced by the larger MNP. Upon reaching the end of the channel at position (**3**), the larger of the two particles has traversed past the midline of the channel (*y* = 0) and will therefore be collected in Fraction 2. The smaller particle remains above *y* = 0 and will be collected in Fraction 1.

**Figure 2:**
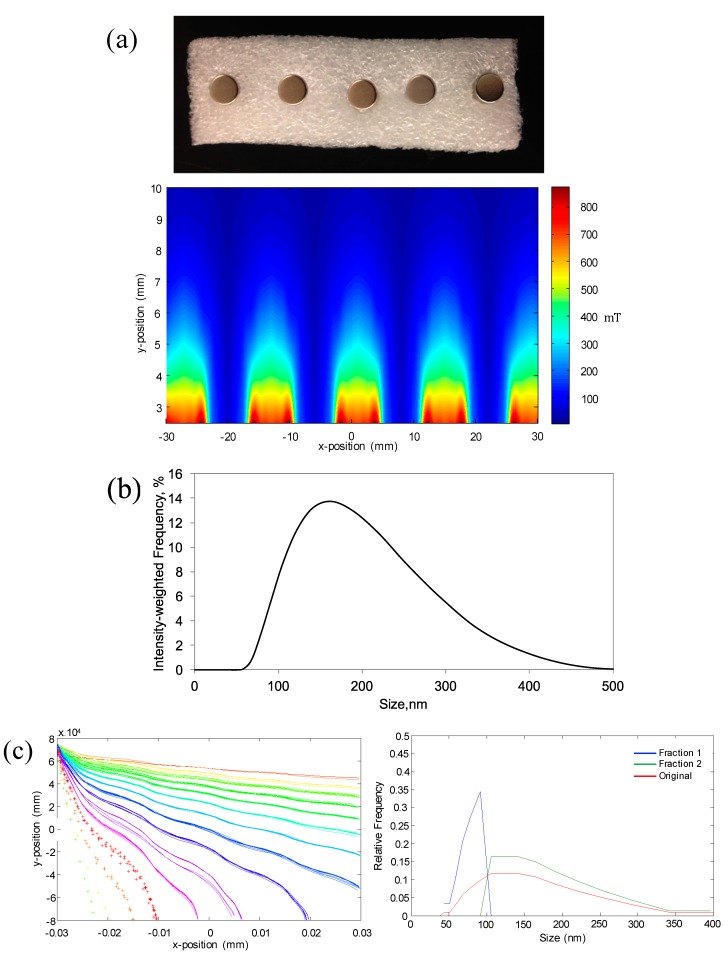

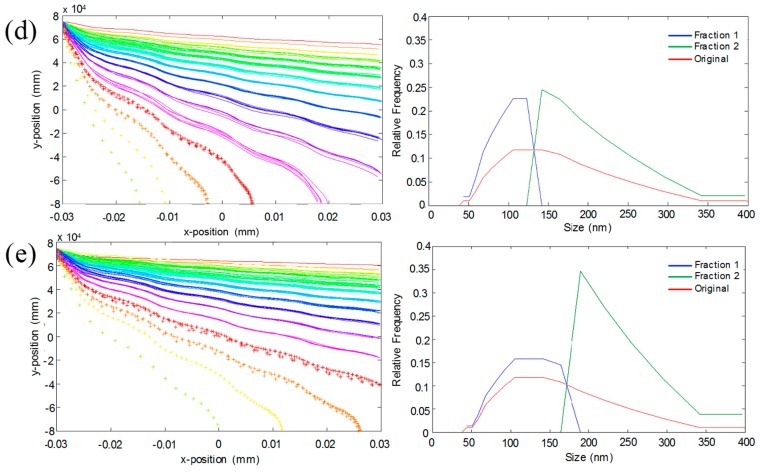
(**a**) Digital image of the sequence of magnets used for the simulation and validation experiments and the magnetic field map generated, using data from the manufacturer, for a series of five non-interacting ¼′′ diameter cylindrical magnets; (**b**) DLS size distribution of original iron oxide nanoparticle suspension; Predicted particle trajectories and resulting size distributions for original MNP (red), Fraction 1 (blue) and Fraction 2 (green) for magnet distances of (**c**) 7.5 mm; (**d**) 10 mm; and (**e**) 11.5 mm. Particle trajectory data sets based on MNP size of −44, −51, −59, −68, −79, −91, −106, −122, −142, −164, −190, −220, +255, +295, +342 and +396 nm.

A Matlab simulation was developed to study the feasibility of separating magnetic nanoparticles of sizes between 50 and 400 nm using the proposed approach. The simulation was based on a proposed experimental design that included 1.6 mm I.D. tubing of length 60 mm running parallel to a magnet, as shown in Figure 1. A Y-split at the end of the tubing (*x* = 60 mm) facilitated separation of MNPS based on their *y*-position. If the final *y*-position of a particle was greater than zero (center of the channel is at *y* = 0) then it was considered to be in Fraction 1, while those at or below the line were considered to be in Fraction 2. The magnetic field was derived from a series of five ¼′′ diameter × ¼′′ length cylindrical neodymium magnets (Cat No.: D44-N52, K & J Magnetics, Pipersville, PA, USA) spaced 7.5 mm apart, as shown in Figure 2a. The magnetic flux density map (Figure 2a) was generated using data provided by the manufacturer and assuming non-interacting magnets.

#### Simulation of Particle Trajectories

Particle trajectories were predicted by force balance, to determine acceleration and velocity, on a single particle at some coordinate ($\;x_{MNP}\left(t\right),\;\;y_{MNP}\left(t\right)$). The new position of the particle, after a time-step *dt*, was determined using the following equations:

(6) $$x_{MNP}\left(t+dt\right)=x_{MNP}\left(t\right)+\;L_{Dx}+\left[\frac{v_{x}\left(t\right)+v_{x}\left(t+1\right)}{2}\right]dt$$

(7) $$y_{MNP}\left(t+dt\right)=y_{MNP}\left(t\right)+\;L_{Dy}+\left[\frac{v_{y}\left(t\right)+v_{y}\left(t+1\right)}{2}\right]dt$$

where $L_{Dx}$ and $L_{Dy}$ refer to the diffusion length in the *x* and *y*-direction, respectively, and $v_{x}$ and $v_{y}$ are the respective velocities in the *x* and *y*-directions. At each time step, the Brownian diffusion of particles was determined by assigning a random fraction of the size-dependent diffusion length, *L_D_*, to the *x*- and *y*-directions.

To mimic the injection of particles through a small capillary tube, the model initially located MNPs at randomly generated distances no greater than 100 μm from the wall opposite the magnets. Each simulation run consisted of 100 nanoparticles, with the particle size distribution determined by data obtained using DLS (Figure 2b).

Using this model, it was predicted that magnetic nanoparticles within the size range of interest could indeed be manipulated in a size-dependent manner using flow through a magnetic field. Three conditions, with the tubing placed 7.5, 10.0, and 11.5 mm from the magnet pole face, were simulated with the fluid velocity and viscosity kept constant at 0.018 m/s and 1.005 mPa·s, respectively. This spacing was predicted to give three distinct size separations, as seen in Figure 2c–e. At a magnet distance of 7.5 mm, a majority of the nanoparticles with sizes between 50 and 100 nm would be in Fraction 1, while larger sized particles will be collected in Fraction 2. Comparatively, at a distance of 11.5 mm it is predicted that nanoparticles with sizes between 50 and 150 nm will be collected in Fraction 1 and the majority of nanoparticles between 200 and 400 nm will end up in Fraction 2. Based on these results, we then attempted to validate our model by replicating the simulation conditions experimentally.

### 3.2. Experimental Validation of the Mathematical Model

**Figure 3:**
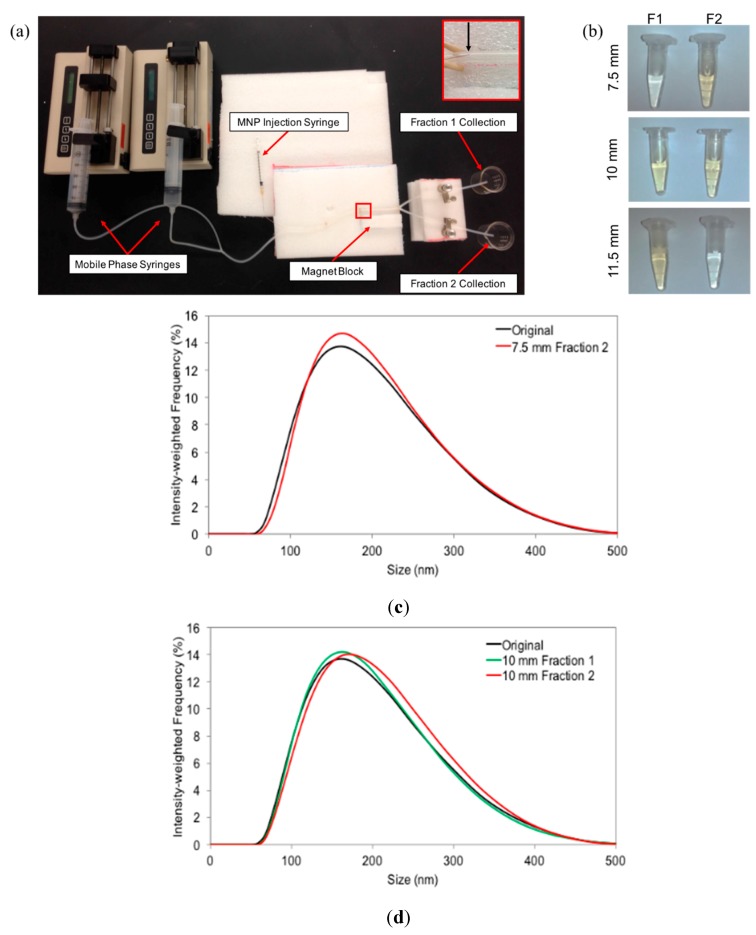

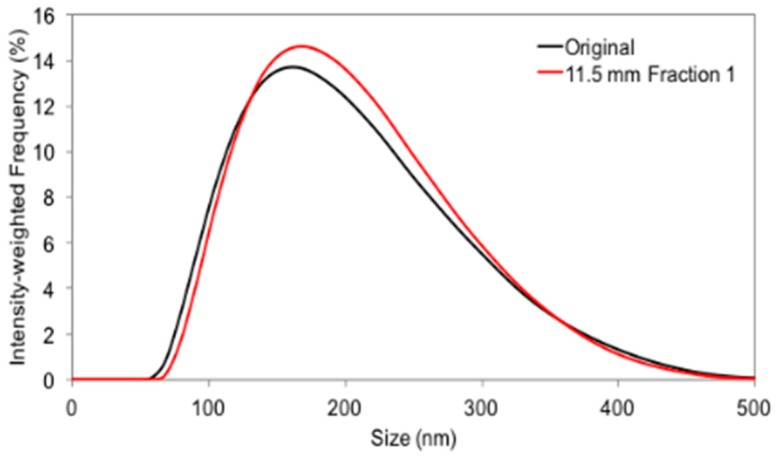
(**a**) Digital image of magnetic separation prototype developed to replicate the conditions of the MATLAB simulation; (**b**) Digital image of Fraction 1 (**left**) and Fraction 2 (**right**) samples collected from the magnet distances of, from top to bottom, 11.5, 10, and 7.5 mm; (**c**–**e**) Size distributions of the obtained samples using distances of 7.5, 10, and 11.5 mm compared to that of the original MNP suspension.

A magnetic separation prototype, as seen in Figure 3a, was developed to reproduce the conditions of the simulation. As with the mathematical model, 1.6 mm I.D. tubing was used to replicate the 2D channel and 100 μm I.D. tubing was used to inject MNPs into the mobile phase at the wall opposite the magnets. Similar to conditions used in the simulation, the magnetic block was placed at distances of 7.5, 10, and 11.5 mm away from the center of the tubing, and a steady fluid flow velocity of 0.018 m/s was maintained using two syringe pumps.

Repeating the simulated runs with the experimental setup, we obtained the samples pictured in Figure 3b. A clear visual difference was observed in the samples taken from Fraction 1 (left column) and Fraction 2 (right column) for the three runs that seemed to coincide with the predicted behavior. For example, in the case of the magnets placed 11.5 mm from the center of the tube (pictured top), it was predicted that a majority of the particles would be found in Fraction 1, with only the larger particles (>200 nm) being found in Fraction 2. Images in Figure 3b seem to agree with this result with the concentration in Fraction 1 apparently higher than that of Fraction 2, as evidenced by the increased coloration. Analysis of the samples using DLS, however, showed that all samples having sufficient particle concentration for measurement (*i.e.*, the colored samples) possessed size distributions nearly identical to that of the original solution, as seen in Figure 3c–e. Samples not showing the yellow coloration (*i.e.*, Fraction 2 for 11.5 mm and Fraction 1 for 7.5 mm) were found to be too dilute to obtain a reliable DLS measurement.

As mentioned, the MATLAB simulation was created as a simple model of the proposed system and it therefore did not account for particle-particle interactions and used a system of magnets assumed to be non-interacting for simplicity. In reality, interactions between the magnets would certainly be present and would alter the magnetic forces experienced by MNPs. Additionally, it is well established that increasing particle concentrations lead to a decrease in inter-particle distance and an increase in particle–particle interactions [33]. In this study, the concentration of MNP suspension injected into the mobile phase was significantly concentrated to ensure final concentrations obtained were appropriate for DLS analysis. This resulted in the particles behaving as a ferro-fluid rather than individual particles and limited size-specific separation. Repetition of these studies at lower MNP concentrations, where particle-particle interactions would be minimized, on the other hand, yielded samples too dilute for characterization. Therefore, it was concluded that magnetic separation of particles suspended in a flow field is unlikely to produce the desired separation on a reasonable scale using this approach.

### 3.3. Successful Size-Selective Elution of Iron Oxide Nanoparticles from an Applied Magnetic Field

Due to the failure of the initial experiments, we then reversed the approach and used flow fields to elute MNPs that were held by a magnetic field. Since the magnetic force is dependent on nanoparticle size, the force required to counteract the magnetic force on two differently-sized MNPs will be lower for the smaller particle. In this case, the counteracting force is the fluid drag force, which is modulated by adjusting the flow rate of the mobile phase.

**Figure 4:**
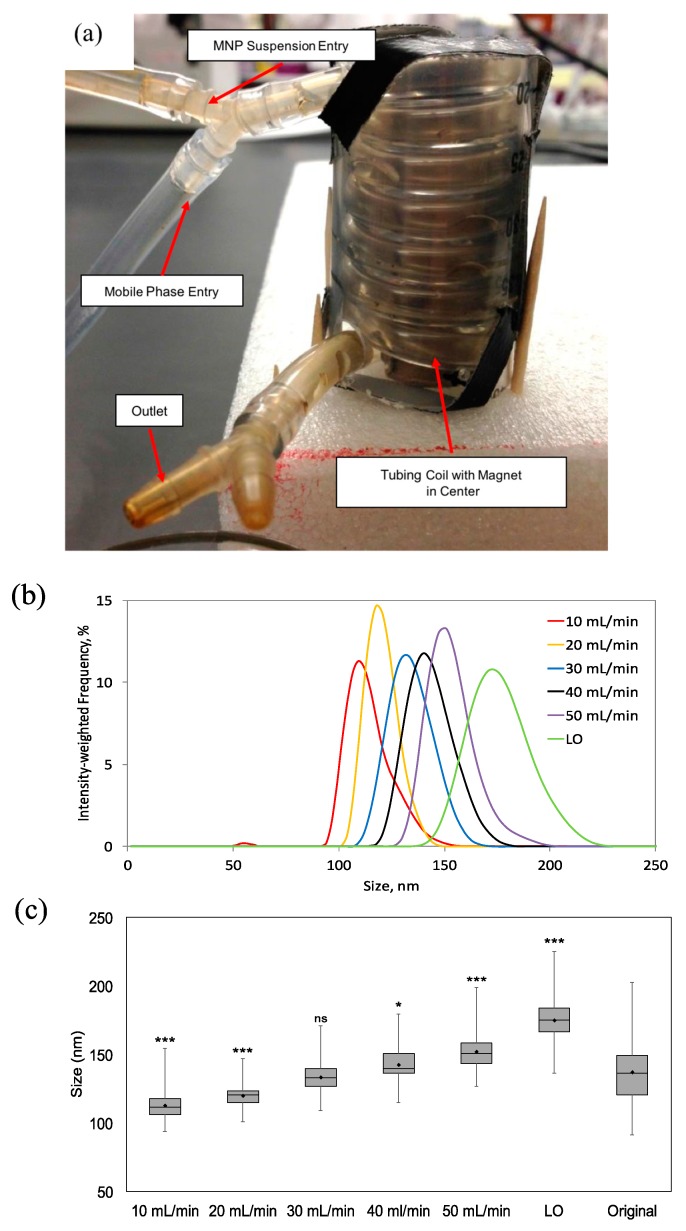
(**a**) Digital image of MagCoil magnetic separation prototype composed of 1/8′′ I.D. tubing wrapped around the length of a 2′′ diametrically magnetized cylinder encased in a plastic column for stability; (**b**) Average size distributions obtained using the MagCoil prototype and flow rates of 10, 20, 30, 40, and 50 mL/min, as well as the flushed particles (LO) calculated from the results of three separate experimental runs; (**c**) Box-and-whisker plot comparing the size distributions obtained using the MagCoil, at the varying flowrates, to the original size distribution. Horizontal lines indicate the mean diameter (nm), while the bar indicates standard deviation, and the vertical line the range. Significance determined using a two-tailed, two sample *t*-test (*n* = 90; *** *p* < 0.001; * *p* < 0.05; ns—not significant). The large sampling size of DLS (>100 k particle counts/s) leads to a statistically significant result between samples that would seem to be identical otherwise.

This was accomplished by creating a simple experimental setup, called the MagCoil, composed of a 18.5′′ length of 1/8′′ I.D. tubing wrapped around the entire 2′′ length of a Grade N42 diametrically magnetized neodymium cylinder (Model No.: ND039-0, Applied Magnets, Plano, TX, USA), as seen in Figure 4a. An inlet for both the MNP suspension and mobile phase were included at the top and a single outlet at the bottom was used for the collection of samples.

After filling the tubing with nanoparticle suspension and allowing them to reach a steady state level of accumulation at the inner wall of the tubing, an initial low flow rate of 0.25 mL/min was introduced to wash away any nanoparticles remaining suspended. DLS measurements of the recovered nanoparticles showed a size distribution similar to the original suspension of nanoparticles (data not shown). A series of increasing flow rates from 10 to 50 mL/min was then applied and samples collected using each of the flow rates for characterization. “Left over” particles, labeled “LO”, that remained in the MagCoil after application of the highest flow rate were then released by removing the magnet and again applying the 50 mL/min flow rate. All experiments were performed in triplicate and the resulting DLS measurements, shown in Figure 4b, are presented as the mean of three experiments. Figure 4c shows the mean hydrodynamic diameter, as well as the standard deviation, range and statistical significance, for each of the size distributions separated out of the original MNP suspension. Samples collected at flowrates of 10, 20, 40 and 50 mL/min and the LO sample, all were statistically different (at least *p* < 0.05) from the original suspension. While the 30 mL/min sample (133 ± 10 nm) could not be distinguished from the original sample (137 ± 21 nm), it should be noted that the separated sample has a four-fold smaller variance (σ^2^). It should also be noted that while the DLS technique typically “counts” more than 100 k samples/s for several seconds, an *n*-value of 90 was used for statistical analysis.

**Figure 5:**
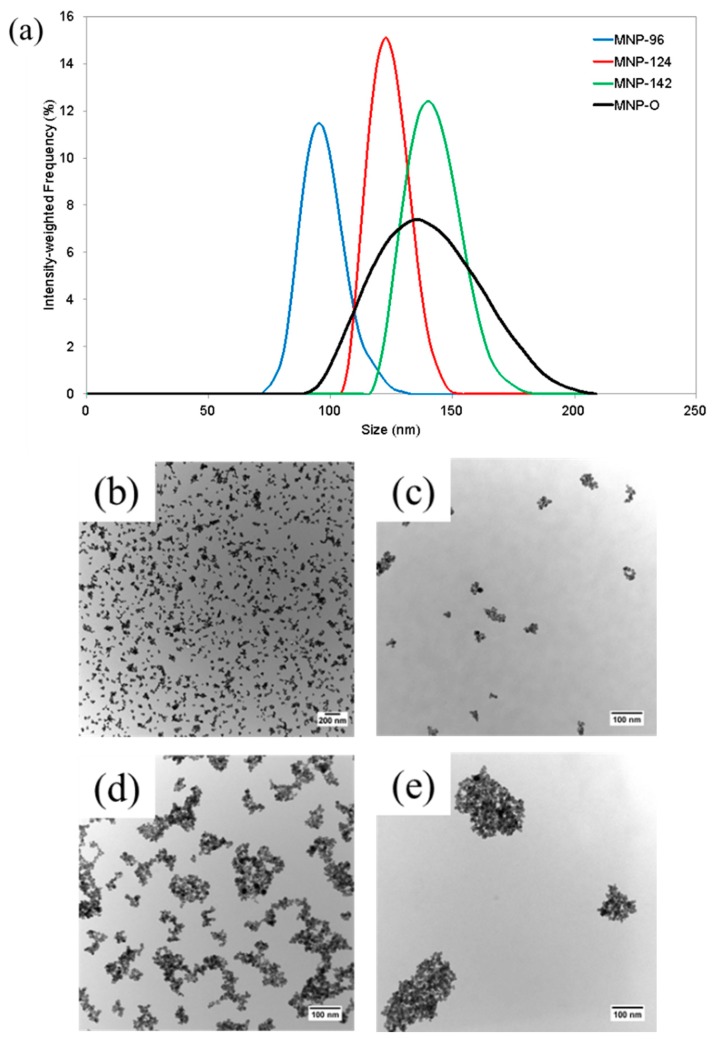
(**a**) Size distributions obtained from the original nanoparticle suspension (MNP-O) at flow rates of 10 mL/min (MNP-96), 40 mL/min (MNP-124), and LO (MNP-142); (**b**) Representative TEM image at 12.5 K magnification of original nanoparticle suspension, MNP-O, before separation; (**c**–**e**) Representative TEM images of MNP-96, MNP-124, and MNP-142 particle samples, respectively, at 85 K magnification.

To confirm the DLS measurements with TEM, samples were again collected with applied flow rates of 10, 40 mL/min, and LO. As shown in Figure 5a, DLS analysis of samples collected from the 10, 40 mL/min, and LO applied flow rates were found to be of size 96.3 ± 9.0, 123.6 ± 7.9, and 141.5 ± 10.8 nm, respectively. These samples were labeled as MNP-96, MNP-124, and MNP-142, respectively. TEM analysis was then performed to both confirm the occurrence of size-dependent separation of the original MNP suspension and for comparison with the obtained DLS results. Figure 5b shows a TEM image of the original suspension, designated as MNP-O. The average size of the original suspension was found to be 75.4 ± 47.7 nm using TEM compared to 137.2 ± 20.8 nm determined using DLS. It is important to note that apparent discrepancy in size is because TEM measurements provide the size of the core diameter while DLS measures the hydrodynamic diameter, which includes the core, surface coating, and any bound solvent. Representative images of each MNP-96, MNP-124, and MNP-142 are given in Figure 5c–e. Analysis of the TEM images showed the average core diameters to be 62.6 ± 27.2, 80.7 ± 45.1, and 104.6 ± 62.3 nm, respectively. A comparison of the measured sizes using both TEM and DLS are given in Table 1.

**Table 1:** Comparison of average hydrodynamic diameters measured using DLS and average core diameters determined using TEM for MNP-O, MNP-96, MNP-124, and MNP-142 distributions.

Sample	DLS (nm)	TEM (nm)
MNP-O	137.21 ± 20.8	75.4 ± 47.7
MNP-96	96.3 ± 9.0	62.6 ± 27.2
MNP-124	123.6 ± 7.9	80.7 ± 45.1
MNP-142	141.5 ± 10.8	104.6 ± 62.3

### 3.4. Size-Dependent Relaxometric Properties of MNP Suspensions

The transverse relaxation time (*T*_2_), which is the decay constant for the magnetization vector ($M_{t}$) perpendicular to the applied magnetic field in MRI, was used to characterize the magnetic properties of fractionated particles obtained using the mFFF approach. Tested samples included the original unseparated suspension (MNP-O; hydrodynamic diameter 137.2 ± 20.8 nm) and separated particles, designated MNP-95 and MNP-151, possessing average diameters of 94.8 ± 7.7 and 151.2 ± 11.2 nm, respectively. A series of dilutions were made of each sample and the relaxivities ($R_{2}=1/T_{2}$) of each sample determined by fitting of MRI data to the equation:

(8) $$M_{t}=M_{0}\;e^{-t/T_{2}}$$

**Figure 6:**
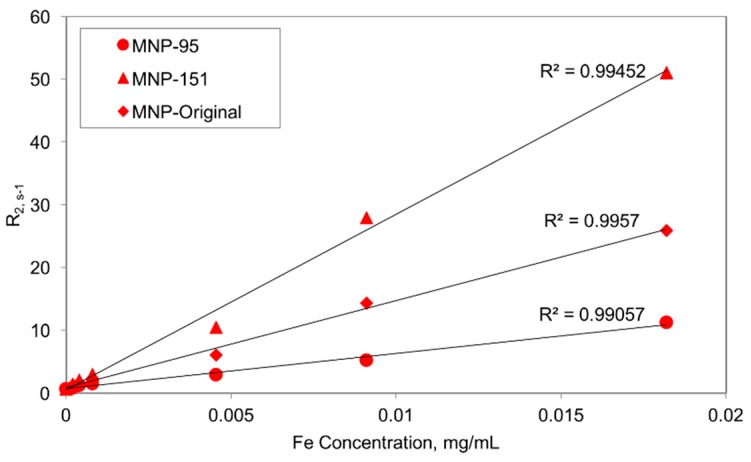
Transverse relaxivity R_2_ values for the MNP-95, MNP-151, and MNP-O distributions with respect to iron concentration. R^2^ values determined using linear trend line.

Preliminary data, shown in Figure 6, indicates that as the average hydrodynamic diameter of the MNPs increased, the transverse proton relaxation time also increased. Considering the inverse relaxation times at the highest concentration used, the R_2_ values increased from 11.2 to 51.05 s^−1^ as the MNP size increased from 95 to 151 nm. This size-dependent behavior is similar to that previously reported for magnetic particles of hydrodynamic sizes less than 100 nm [34]. Additionally, it is interesting to note that the *R*_2_ values of the MNP-O samples, which contain particles of the same size as both MNP-95 and MNP-151, as well as sizes between the two distributions, were consistently between the *R*_2_ values of MNP-95 and MNP-151. For example, the R_2_ value for the MNP-O sample with an iron concentration of 0.009 mg/mL was determined to be 14.3 s^−1^ compared to the values of MNP-95 and MNP-151 at the same concentration of 5.2 and 27.87 s^−1^, respectively.

## 4. Experimental Section

### 4.1. Materials

fluidMAG-D (starch-coated magnetite (Fe_3_O_4_)) iron oxide nanoparticles (75 mg/mL) were obtained from Chemicell^®^ GmBH (Berlin, Germany). Succinimidyl polyethylene glycol (mPEG-NHS) of molecular weight 5 kDa was obtained from Nanocs (New York, NY, USA). Dimethyl sulfoxide ((CH_3_)_2_SO, 99.9%) was obtained from BDH Chemicals. Epichlorohydrin (C_3_H_5_ClO, 99%) was obtained from Alfa Aesar (Ward Hill, MA, USA). Sodium hydroxide (NaOH, 97%) was obtained from BDH chemicals. Ferrozine iron reagent, monohydrate was obtained from J.T. Baker (Center Valley, PA, USA). Neocuproine hydrochloride monohydrate (C_14_H_12_N_2_·HCl·H_2_O, 99%) was obtained from Acros. Ammonium acetate, ACS (CH_3_COONH_4_, 97% min) was obtained from Alfa Aesar. l-ascorbic acid (C_6_H_8_O_6_) was obtained from BDH. Iron standard solution (1.00 mg/L as Fe) was obtained from HACH. Deionized water (DI-H_2_O) was obtained using an ELGA PURELAB Flex water purification system.

### 4.2. Surface Modification of Iron Oxide Nanoparticles

The IO-MNPs were cross-linked, aminated, and PEGylated according to previously established methodology [35]. First, 2 mL of MNP suspension (42 mg/mL) was incubated with 2.6 mL 6 M NaOH for 15 min. Epichlorohydrin (1.3 mL) was then added and the mixture incubated for 24 h at 25 °C with shaking. After incubation, the solution was thoroughly dialyzed against DI-H_2_O using a 8–10 kDa MWCO Float-A-Lyzer^®^ G2 dialysis device (Spectrum Laboratories, Inc., Rancho Dominguez, CA, USA). The purified product was then incubated with 2 mL of concentrated NH_4_OH (30% ammonia) for a period of 24 h at 25 °C with shaking. The aminated-MNP suspension was then sufficiently dialyzed against DI-H_2_O and the final product was concentrated using a Sphero™ Fleximag Separator (Spherotech, Lake Forest, IL, USA).

The PEGylation of the MNPs was achieved by utilizing NHS chemistry. First, 15 mg of mPEG-NHS was dissolved in a mixture of 320 μL of DMSO, 320 μL of DI-H_2_O, and 320 μL of pH 8 phosphate buffer. 320 μL of aminated-MNP solution was then added and the mixture was incubated at 25 °C with shaking. At the completion of incubation, the solution was diluted to ~7 mL with DI-H_2_O, placed on the magnetic separator, and then subjected to several washes with fresh DI-H_2_O. After washing, the PEG-MNP solution was diluted to the final desired concentration.

### 4.3. Characterization of MNPs

*Dynamic Light Scattering (DLS)—*Dynamic light scattering was used to measure the intensity-weighted size (hydrodynamic diameter) distribution of nanoparticles. Measurements were taken in triplicate using a ZetaSizer Nano ZS90 sizing instrument (Malvern, Worcestshire, UK).

*Transmission Electron Microscopy (TEM)—*A Zeiss EM 10 TEM operating at a voltage of 60 kV was used to determine size distributions of MNPs. TEM samples were prepared by placing a single drop of a MNP solution onto a carbon type B, 300 mesh grid. The grid was then placed in a petri dish and allowed to dry at ambient conditions. Size distributions were obtained using ImageJ software to size a sufficient number of MNPs from multiple TEM images taken of each sample.

*Iron Content Assay—*The iron content of MNP solutions was determined using a ferrozine assay. Briefly, a 200 μL dilution (typically 1:1000) of the MNP sample was obtained in combination with 1 M HCl. 230 μL of KMnO_4_/HCl was added to the sample and mixed via pipette. The KMnO_4_/HCl solution was made by mixing equal volumes of 4.5% *w*/*v* KMnO_4_ with 1.4 M HCl. The mixture was then incubated for 2 h at 60 °C followed by a 10 min cooling period. The sample was then mixed and transferred to a well plate via two 180 μL aliquots. Thirty microliters of prepared ferrozine solution was then added to the samples, mixed, and incubated at ambient conditions for 30 min. The prepared ferrozine solution was composed of 6.5 mM ferrozine, 6.5 mM neocuprine, 2.5 M ammonium acetate, and 1 M ascorbic acid dissolved in DI-H_2_O. The absorbance of the samples at 550 nm was then measured using a SpectraMax i3 plate reader (Molecular Devices, Sunnyvale, CA, USA). Standard curves were created using an iron standard solution.

### 4.4. Magnetic Separation Prototype Operation

The magnetic separation prototype was created using styrofoam to create a block for the five cylindrical magnets and a platform for the tubing. 1/16′′ I.D. silicone tubing (VWR) was used to replicate the 2D channel in the simulation. 100 μm flexible fused silica capillary tubing (Molex, Lisle, IL, USA) was used to inject the nanoparticle solution into the mobile phase at the wall opposite the magnets. This tubing was connected to a 1 mL syringe fitted with a 30 G needle via a small section of 0.011′′ ID polyethylene tubing (Clay Adams, Sparks, MD, USA). The mobile phase was supplied by two syringe infusion pumps (KD Scientific, Holliston, MA, USA) in order to control the flow rate of the system.

For a typical run, the desired flow rate of the mobile phase was set to 0.036 mL/s using the syringe pumps and infusion was started. The concentrated nanoparticle suspension was then injected into the mobile phase by applying a small amount of pressure to the syringe plunger. The nanoparticle suspension was continually injected until the desired volume (approximately 0.1 mL) had been run through the magnetic separation prototype. After this, infusion of the mobile phase was terminated and the two obtained fractions were characterized. The whole system was flushed sufficiently with DI water to ensure no cross-contamination between runs.

### 4.5. Field-Flow Fractionation Prototype Operation

For all field-flow fractionation experiments, the desired volume of concentrated nanoparticle suspension was injected into 1/8′′ ID silicone tubing (VWR, Radnor, PA, USA) in the absence of flow from the mobile phase. The nanoparticles were then allowed to collect at the wall of the tubing closest to the magnet for a period of 15 min to allow for a steady-state distribution of particles at the tubing wall. The mobile phase was then introduced at a low initial flow rate using a peristaltic pump (Thermo Fisher Scientific, Waltham, MA, USA) in order to wash away any MNPs that had not collected at the tubing wall. After the entire volume of the silicone tubing was washed, flow from the pump was stopped, the sample volume was removed from the collection vessel for characterization, and then the collection vessel was rinsed with DI water and placed at the outlet. A higher flow rate was then introduced into the system and another sample was collected. This process was repeated for all desired flow rates supplied by the pump. Lastly, MNPs remaining in the tubing were removed by flushing with DI water after removal of the magnetic field.

### 4.6. Relaxometric Property Determination Using MRI

Relaxometry measurements were performed using a Siemens Verio Open-Bore 3T Scanner to determine the transverse proton relaxation times of solutions of iron oxide nanoparticles via spin-echo pulse sequences. For measurements of aqueous MNP solutions, samples were placed in either 0.6 or 1.6 mL plastic microfuge tubes and the tubes submerged in water spiked with copper sulfate (CuSO_4_) to control background noise. The relaxation times of each sample were determined by plotting the magnitude of the measured MR signal at each of the echo times used in the spin-echo sequence and using a curve-fitting MATLAB script.

## 5. Conclusions

For the full potential of magnetic nanoparticles in nanomedicine to be realized, methods must be developed that allow for the distinct control of their physical and chemical properties so that particles may be optimized for specific applications. One of the most important factors that determines the behavior of magnetic nanoparticles *in vivo* is their size; however, current synthesis methods do not allow for sufficient size control for iron oxide nanoparticles of diameters greater than 30 nm. While initial Matlab simulations indicated that size-selective separation could be achieved by using magnetic fields to isolate MNPs from a liquid flow field, subsequent experiments were unable to confirm the results predicted by the model. Since a ferrofluid-type behavior was observed, the discrepancy between the theoretical and experimental results could be due to the assumption that particle-particle interactions were not significant within the model. Further improvements to both the model and the experimental setup are ongoing to validate this approach for the size-selective separation of magnetic nanoparticles. Despite this initial failing, an approach utilizing mechanisms similar to magnetic field-flow fractionation was found to be an effective method for the separation of polydisperse suspensions of iron oxide nanoparticles with diameters greater than 20 nm. While similar methods have been previously used to separate magnetic nanoparticles, there has been very little reported on the separation of particle in the size range of 50–400 nm. TEM and DLS analysis of particles obtained using this approach confirmed that particles of varying size and lower polydispersity can indeed be obtained within this size range.

An advantage of the magnetic separation approach used here is its simplicity and use of basic laboratory equipment, not requiring, for example, the special membranes required for cross-flow FFF. With a simple neodymium magnet, tubing, and a source of variable flow, this approach is easily adoptable to other laboratories. Ongoing studies are further refining the separation efficiency and scaling-up the approach to generate higher concentration of particles required for *in vivo* studies. These studies and further optimization of the system presented in these investigations could allow for the production of very narrow size distributions of magnetic nanoparticles within the size range relevant for biomedical applications in a very simple and economic manner. This could substantially improve the potential for clinical translation of these particles by enabling the fundamental studies necessary to understand the disposition of these particles *in vivo*.
